# Osteochondral Lesion of the Talus Amplifies Plantar Pressure Alterations and Postural Instability in Chronic Lateral Ankle Instability: A Cross‐Sectional Study Based on a Wearable Smart Plantar Pressure System

**DOI:** 10.1002/jfa2.70110

**Published:** 2025-12-17

**Authors:** Ting Zhu, Rui Chen, Haoyang Kang, Fangyuan Ding, Rui Guo, Xiaoming Wu, Dong Jiang

**Affiliations:** ^1^ Department of Sports Medicine Peking University Third Hospital Institute of Sports Medicine of Peking University Beijing China; ^2^ Beijing Key Laboratory of Sports Injuries Beijing China; ^3^ School of Integrated Circuits Tsinghua University Beijing China

**Keywords:** biomechanics, chronic lateral ankle instability, joint degeneration, osteochondral lesion of the talus, plantar pressure

## Abstract

**Background:**

Chronic lateral ankle instability (CLAI) is frequently complicated by osteochondral lesion of the talus (OLT), which accelerate cartilage degeneration due to abnormal stress concentration and may progress to ankle osteoarthritis. However, in vivo biomechanical alterations in patients with concurrent CLAI and OLT remains unclear. This study aimed to characterize plantar pressure and stability deficits during single‐leg stance (SLS) in patients with CLAI with and without OLT compared to healthy controls.

**Methods:**

Eighty‐eight participants were recruited: 29 healthy controls, 30 patients with CLAI, and 29 patients with CLAI + OLT (patients with CLAI and OLT). All participants underwent clinical assessments, including MRI‐based ligament and cartilage evaluations. Plantar pressure parameters (normalized peak force [PF%]) and postural stability metrics (center of pressure [COP] and time‐to‐boundary [TTB]) were collected using a shoe‐integrated sensor system during SLS. Among‐group differences were analyzed using ANOVA and independent *t*‐tests, with additional subgroup analyses based on gender, body mass index (BMI), and generalized joint hypermobility.

**Results:**

Compared to control group, both CLAI (*p* = 0.028) and CLAI + OLT (*p* = 0.001) groups exhibited elevated medial midfoot PF% and patients with CLAI demonstrated higher PF% in third metatarsal region (*p* = 0.015). Patients with CLAI + OLT demonstrated reduced TTB (*p* = 0.032) and greater COP variance (*p* = 0.026) in the anterior–posterior direction. When the two sides were compared, the unaffected side in the CLAI + OLT group displayed lower PF% in the posterior heel (*p* = 0.012) and higher PF% in the fifth metatarsals (*p* = 0.030). Receiver operating characteristic curve analysis identified PF% in the third metatarsal as a moderate diagnostic marker for OLT (AUC = 0.700 and *p* = 0.026). Subgroup analyses revealed that patients with CLAI with males (*p* = 0.047), BMI < 25 (*p* = 0.010), and Beighton scores < 5 (*p* = 0.004) exhibited elevated PF% in the third metatarsal than CLAI + OLT, and the CLAI + OLT group with BMI ≥ 25 showed increased PF% in posterior heel (*p* = 0.043).

**Conclusions:**

Patients with CLAI, particularly those with concomitant OLT, exhibited distinct biomechanical adaptations characterized by medial midfoot overload and impaired anterior–posterior stability. The implementation of early biomechanical screening of third metatarsal pressure in patients with CLAI and tailored rehabilitation for those with male sex, BMI < 25, and Beighton < 5 was necessary to mitigate osteoarthritis progression.

**Abbreviations**
AOFAS: American Orthopedic Foot and Ankle Society
AP: anterior–posterior
ATFL: anterior talofibular ligament
AUC: area under curve
BMI: body mass index
CAI: chronic ankle instability
CFL: calcaneofibular ligament
CLAI: chronic lateral ankle instability
COP: center‐of‐pressure
COPV: center‐of‐pressure velocity
HA region: anterior heel
HP region: posterior heel
M1 region: first metatarsal
M3 region: third metatarsal
M5 region: fifth metatarsal
ML: medial‐lateral
ML region: lateral midfoot
MM region: medial midfoot
MRI: magnetic resonance imaging
OA: osteoarthritis
OLT: osteochondral lesion of the talus
PF: plantar force
ROC: receiver operating characteristic
SLS: single‐leg stance
T1 region: the first phalanx
TTB: time‐to‐boundary

## 1 Introduction

Ankle sprains are among the most commonly occurring musculoskeletal injuries, and 80% of all ankle sprains involve the lateral ligaments including anterior talofibular ligament (ATFL) and calcaneofibular ligament (CFL) [1]. Furthermore, 20%–40% of individuals experience recurrent sprains due to incomplete ligament healing, which may progress to chronic lateral ankle instability (CLAI) [2, 3]. CLAI is primarily characterized by symptoms such as pain, swelling, feelings of instability, recurrent sprains, and functional impairment [4]. Moreover, it is strongly associated with intra‐articular pathologies, including chondral and osteochondral lesions [5, 6]. Studies indicate that these lesions are present in up to 32% of ankles with CLAI [7]. Prolonged abnormal stress distribution, particularly localized stress concentration within the ankle joint, could accelerate cartilage degeneration and potentially lead to osteochondral lesion of the talus (OLT), and may ultimately contribute to the development of ankle osteoarthritis (OA), affecting the normal life and work of patients [8].

Abnormal or excessive mechanical loading is closely related to the occurrence and progression of OLT. Although most studies have explored the functional relationship between cartilage and mechanical loading through cell, animal, or cadaveric experiments, these approaches fail to clarify the relationship between overall joint loading and cartilage degeneration in patients [9, 10]. Therefore, in vivo clinical biomechanical research is essential for understanding abnormal joint mechanics and postural control as well as optimizing interventions, such as rehabilitation training and assistive devices, to regulate joint loading and slow OA progression. Existing studies have identified biomechanical characteristics in patients with CLAI. Mortezanejad et al. demonstrated increased sagittal plane stiffness in patients with CLAI during a gait initiation task as evidenced by decreased center of pressure velocity and peak torque, along with prolonged postural adjustment time, using integrated motion capture and surface electromyography [11]. Hou et al. revealed significant deficits in plantar flexor and eversion strength, particularly in females using Biodex isokinetic testing [12]. Although some studies have begun investigating the biomechanical alterations in patients with CLAI with concomitant OLT [13, 14], current research remains primarily focused on OLT surgical treatments and clinical outcomes [15, 16], with limited investigations into the biomechanics of this specific patient group.

Plantar pressure measurement is a valuable biomechanical tool for assessing ankle joint function and motor control, with portable pressure insole systems providing a practical and flexible approach for evaluating natural movement across various tasks and environments [17, 18]. There is increasing evidence that patients with chronic lateral ankle instability (CAI) exhibit significant changes in plantar pressure distribution and center of pressure (COP) characteristics during tasks such as walking, running, and single‐leg standing. For example, Wan et al. reported that patients with unilateral CAI show lateralized plantar load distribution during the support phase of walking, with prolonged support time and increased COP velocity [19]. Morrison et al. observed that patients with CAI manifest increased lateral pressure in the rearfoot and forefoot together with a laterally directed COP trajectory during barefoot running [20]. Furthermore, Abe et al. using force plates and head/foot accelerometry, identified asymmetric COP patterns during single‐leg stance (SLS) in individuals with a history of ankle sprains, further highlighting their postural instability [21]. Although many studies have explored the plantar pressure characteristics of patients with isolated CAI, there is still a lack of research on the biomechanical characteristics of patients with CAI combined with OLT. Conventional movement analyses, such as level walking, may not be sufficiently challenging to uncover subtle impairments in patients with CLAI [22]. Although, SLS presents a more demanding condition, allowing for the detection of underlying postural and biomechanical deficits [23]. Therefore, identifying plantar pressure characteristics during SLS in patients with CLAI and OLT enables a more accurate evaluation of the relationship between postural control, stability, and disease progression, thereby providing a stronger scientific foundation for clinical treatment strategies.

In the present study, 88 participants were recruited, including 29 healthy subjects, 30 patients with CLAI, and 29 patients with concomitant CLAI and OLT. Normalized peak force, COP, and time‐to‐boundary (TTB) during SLS were measured using a portable shoe‐integrated detection system. The purpose of this study was to analyze the plantar pressure characteristics during single‐leg stance of patients with CLAI and those with both CLAI and OLT, comparing them with healthy individuals. We hypothesized that plantar pressure distribution in both groups was distinct from that in healthy controls, and this difference might be prominent in those with both CLAI and OLT. The findings of this work may enhance the understanding of the pathogenesis of CLAI with concomitant OLT and identify potential clinical biomechanical targets for more effective treatment.

## 2 Methods

### 2.1 Study Design

**FIGURE 1:**
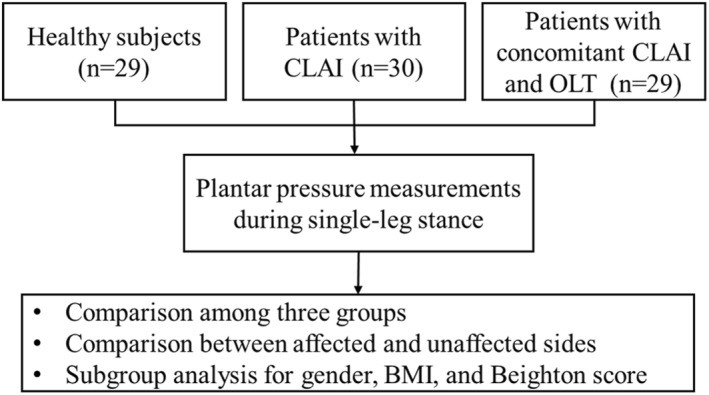
A flow diagram of the study.

This was a cross‐sectional study. In the present study, 88 participants were recruited. The basic characteristics of participants were obtained firstly. Next, patients underwent clinical examinations and magnetic resonance imaging (MRI) examination and were divided according to the damage of ankle osteochondral into a CLAI group and a concomitant CLAI and OLT (CLAI + OLT) group. Finally, the plantar pressure characteristics during SLS were tested by a novel smart shoe‐integrated sensor system. Normalized peak force, COP, and TTB were measured. The experimental flow was shown in Figure 1.

### 2.2 Participants

A power analysis using G*Power (Version 3.1.9.2; Kiel, Germany), based on plantar pressure data from a previous study, determined that 16 participants per group were needed (*α* = 0.05, power = 0.80, and effect size *f* = 0.465). To accommodate planned exploratory subgroup analyses, the target sample size was increased to 30 participants per group. All of the patients were recruited from the outpatient sports medicine clinic of Peking University Third Hospital (Beijing, China), and healthy controls were recruited from surrounding communities via advertisement. All participants were screened against predefined inclusion and exclusion criteria before enrollment.

**FIGURE 2:**
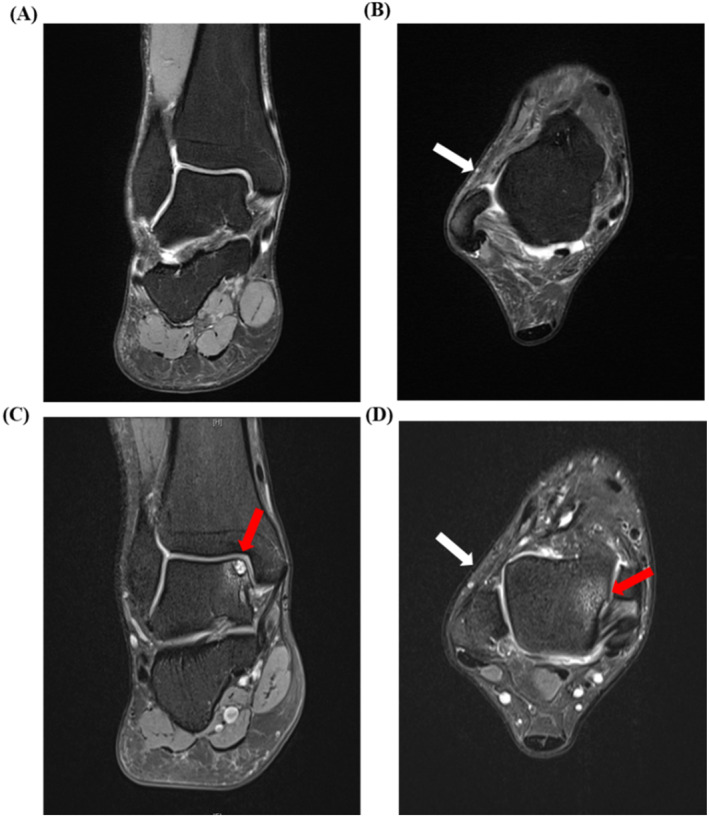
MRI images of typical patients from the CLAI group and the CLAI + OLT group: (A) coronal MRI image of a typical patient with CLAI showing no talar cartilage lesions; (B) transverse MRI image of a typical CLAI patient with CLAI showing anterior talofibular ligament injury. White arrow: anterior talofibular ligament injury; (C) coronal MRI image of a typical patient with CLAI + OLT showing talar cartilage lesions. Red arrow: OLT; and (D) transverse MRI image of a typical patient with CLAI + OLT showing both anterior talofibular ligament injury and talar cartilage lesions. White arrow: anterior talofibular ligament injury, red arrow: OLT. CLAI + OLT, patient with chronic ankle instability and osteochondral lesion of the talus.

In accordance with the International Ankle Consortium guidelines [24], the inclusion criteria of patients were as follows: (1) age between 18 and 55 years; (2) unilateral ankle sprain confirmed by an experienced foot and ankle specialist through a positive anterior drawer and/or inversion stress test, with MRI evidence of chronic injury to the ATFL and/or CFL, whereas the contralateral ankle remained healthy; (3) a history of at least one significant unilateral ankle sprain, with the first sprain occurring no < 1 year before enrollment and the most recent injury happening more than 6 months prior to enrollment; (4) history of the affected ankle “giving way” more than twice and/or feelings of instability during daily or sports activities; and (5) a Cumberland Ankle Instability Tool score < 24. Exclusion criteria of patients were as follows: (1) bilateral CLAI; (2) foot deformities, including flat feet, high arched feet, hallux valgus, Charcot foot, or hindfoot deformities; (3) any previous lower‐limb surgery or fractures; (4) presence of ankle osteoarthritis on MRI, defined as diffuse cartilage thinning, subchondral bone sclerosis, or osteophyte formation; (5) neuromuscular diseases, or other motor system disorders that may impair normal gait; and (6) unwilling to sign informed consent. Based on MRI images, patients were divided into two groups: those without concurrent OLT (Figure 2A,B) and those with concurrent OLT (Figure 2C,D).

The healthy control group was selected based on the following criteria: (1) age range of 18–55 years and (2) had no history of ankle sprains. Exclusion criteria of healthy subjects were as follows: (1) history of significant lower limb injuries, including hip, knee, ankle, or musculoskeletal injuries; (2) diseases of the neuromuscular system; and (3) unwilling to sign informed consent. All participants were thoroughly briefed on the procedures before the study and signed informed consent forms prior to data collection. This study received approval from the Medical Ethics Committee of Peking University Third Hospital (M2024712).

### 2.3 Equipment

**FIGURE 3:**
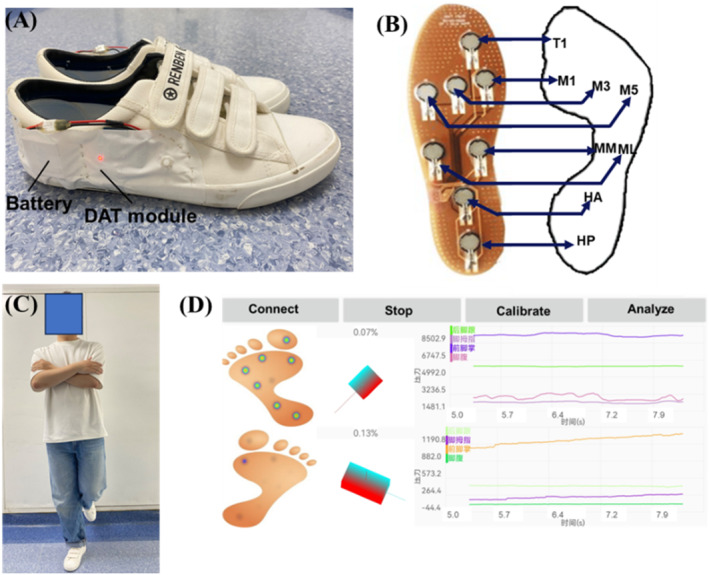
The user stand unilaterally with the pressure detection shoes whereas data were obtained and transformed into a smartphone in time: (A) custom‐made plantar pressure testing shoes used in the present study; (B) schematic of the insole pressure sensor layout, illustrating the correspondence between each sensor and anatomical foot regions; (C) demonstration of the single‐leg stance testing; and (D) the real‐time plantar pressure acquisition interface.

Plantar pressure data were collected at a sampling rate of 100 Hz using a real‐time, low‐cost, and shoe‐integrated sensor system (Figure 3) [25]. The system's reliability and validity have been clinically validated, and it has received a European Union approved Conformity Marking (CE) (CE Certificate Number: B‐S210134605) [25]. Eight force‐sensitive resistor sensors were strategically embedded at key anatomical locations: the first phalanx (T1), first metatarsal (M1), third metatarsal (M3), fifth metatarsal (M5), medial midfoot (MM), lateral midfoot (ML), anterior heel (HA), and posterior heel (HP) [17]. To ensure reliable data collection, the sensors were appropriately calibrated before use. Real‐time data acquisition was facilitated by a flexible printed circuit board connected to a compact Data Acquisition and Transmission module, comprising an analog front‐end, analog‐to‐digital converter, microprocessor, and Bluetooth module. The system offered significant advantages for clinical applications, including portability, ease of use, real‐time monitoring, and high accuracy. To maintain optimal sensor contact and preserve natural motion state patterns, plantar pressure detection shoes were provided in various sizes (UK4–UK10) [17].

### 2.4 Imaging Measurement

A Signal HDxt 3.0‐T MRl system (GE Healthcare) with an eight‐channel ankle joint surface coil was applied in this study. The coronal, sagittal, and transverse images in the proton density spin‐echo scans were used to perform the measurement in the picture archiving and communication system. The measurement methods of the length and width of the ATFL and the area and volume of injured OLT were based on previous studies.

#### 2.4.1 Length of the ATFL

The length of the ATFL was measured through transverse MRI images that show the full length of the ligament, represented by the distance from the anterior and inferior borders of the fibula to the talar neck [26].

#### 2.4.2 Width of the ATFL

The width of the ATFL was measured at its midpoint, using the same MRI slice image as the one used to measure the length of the ATFL [27].

#### 2.4.3 The OLT Area and Volume

The coronal and sagittal proton density‐weighted MRI image showing the most extensive lesion was used for OLT measurement. Maximal coronal and sagittal lengths were defined as the medial‐lateral and anterior‐posterior dimensions, respectively, whereas OLT depth was measured as the greatest distance from the articular surface to the lesion's base. The OLT area and volume were calculated by the following formulas: OLT area = (coronal length/2) x (sagittal length/2) × π. Lesion volume = (coronal length/2) × (sagittal length/2) × (depth/2) × (4π/3) [28, 29].

### 2.5 Clinical Assessment

Basic information, including age, sex, height and weight, course, and duration of the disease, affected side of the patients was recoded. The Beighton score is a 9‐point assessment tool used to evaluate systemic joint laxity and a score > 4 points indicates joint laxity [30]. The American Orthopedic Foot and Ankle Society (AOFAS) is a comprehensive tool designed to assess the function and condition of the ankle and hindfoot. The total score is 100 points, with higher scores reflecting better ankle function [31].

### 2.6 Plantar Pressure Analysis During Single‐Leg Stance

#### 2.6.1 Single‐Leg Stance

After standing practice trials, each participant wore the plantar pressure detection shoes and completed three 10‐s single‐leg stance trials (Figure 3). During each trial, participants were instructed to remain as still as possible, with their arms crossed over their chest and looking forward. The nonsupporting leg flexed at approximately 45° at the knee and 30° at the hip. Participants were tested on both legs separately, with the unaffected leg tested first for two patients groups and the dominant leg tested first for the healthy control group (the preferred leg to kick a soccer). The values of the 3 trials were averaged and used for the analysis [32].

#### 2.6.2 Characteristics of Plantar Load Assessment

The normalized peak force (PF%) in each subregion, center‐of‐pressure (COP), and time‐to‐boundary (TTB) parameters were evaluated. For plantar pressure analysis, peak force was calculated and normalized to body weight to minimize inter‐individual variability associated with body mass and anthropometric factors [33]. PF% provided a direct and comparable measure of plantar load distribution and was calculated as follows: $\text{PF}\left(\%\right)=\frac{\text{Peak}\;\text{force}\;\left(N\right)}{\text{Body}\;\text{weight}\;\left(\text{kg}\right)\times g}\times 100\%$, where *g* represented the gravitational acceleration [34, 35, 36]. COP and TTB analyzed balance and stability from spatial and temporal dimensions, respectively. For each measurement, the instantaneous position of the COP was used to determine the distance to the medial‐lateral (ML) and anterior–posterior (AP) edges of the foot [37]. TTB was computed by dividing this distance by the corresponding instantaneous COP velocity at each time point, with lower TTB values indicating greater postural instability. COP parameters included range and variance in both ML and AP directions, whereas TTB parameters comprised absolute, mean, and standard deviation (SD) values for both directions [38]. The dominant leg of healthy participants was compared with the affected side of both patient groups [39, 40].

#### 2.6.3 Raw Data Processing

To provide a detailed visualization for biomechanical analysis, the plantar pressure maps for both feet were created. Plantar pressure maps for both feet were generated to provide detailed biomechanical visualization. Raw sensor readings were neither removed nor altered. A foot‐contour–based geometric mask confined interpolation to the plantar surface. Pressure values were interpolated using a cosine‐based attenuation function within a radius (*d*) of each sensor, with additional sine‐based attenuation near boundaries (*d*_1_) to minimize edge effects. The interpolated maps were smoothed with a 4 × 4 mean filter applied iteratively three times to reduce high‐frequency noise. All steps were implemented in MATLAB (R2023b).

### 2.7 Statistical Analysis

Data normality was assessed using the Shapiro–Wilk test. Continuous variables following a normal distribution were expressed as mean ± SD, whereas categorical data were reported as frequencies. The chi‐squared test was applied to assess differences in sex, affected side, and trauma mechanisms. Plantar pressure metrics (PF%, COP, and TTB) were evaluated using analysis of variance (ANOVA) to compare the healthy and patient groups, and for significant ANOVA results, Bonferroni post hoc test was conducted. Paired *t*‐tests were used to evaluate differences in plantar pressure parameters between the affected and unaffected feet in the two patients group. Receiver operating characteristic (ROC) curve analysis were conducted to evaluate the discrimination ability between the two patients groups. Subgroup analyses were conducted within the CLAI concomitant with the OLT group and the CLAI group based on gender, body mass index (BMI), and Beighton score. All statistical analyses were performed by SPSS (Version 23, Chicago, IL), with statistical significance set at *p* < 0.05.

## 3 Results

### 3.1 Participant Characteristics

Ninety participants who met eligibility criteria were originally recruited, but one healthy subject did not have enough time and one patient with CLAI and OLT failed to complete the SLS assessment. Consequently, 88 participants were included in the final analysis (29 healthy controls, 30 patients with CLAI, and 29 patients with CLAI and OLT). No significant differences were observed in age, sex, height, weight, or BMI among the three groups. Injury characteristics, such as affected side, time since injury, trauma mechanism, Beighton score, and AOFAS score, also revealed no significant differences. MRI evaluations of the ATFL revealed no significant variations in length or width between the CLAI group and the CLAI + OLT group. The baseline characteristics were presented in Table 1.

**TABLE 1:** Baseline characteristics of participants.

Parameters	Control group (*n* = 29)	CLAI (*n* = 30)	CLAI + OLT (*n* = 29)	*p* value
Age (years)	34.47 ± 10.66	30.00 ± 9.95	32.14 ± 9.14	0.226
Gender (male/female, *n*)	23/6	18/12	24/5	0.099
Height (cm)	174.07 ± 5.89	172.93 ± 8.85	174.79 ± 7.88	0.642
Weight (kg)	71.90 ± 12.52	71.90 ± 15.86	77.45 ± 9.90	0.176
BMI (kg/m^2^)	23.66 ± 3.58	23.85 ± 3.77	25.28 ± 2.14	0.119
Injured side (left/right, *n*)		15/15	13/16	0.691
Trauma mechanism, *n*				
Sport injury		16	20	0.135
Falling		13	6	
Other		1	3	
Beighton score		3.67 ± 2.97	2.69 ± 2.70	0.193
AOFAS		82.67 ± 10.04	79.79 ± 11.12	0.302
ATFL length (mm)		17.73 ± 4.02	19.29 ± 4.56	0.168
ATFL width (mm)		3.65 ± 1.41	3.88 ± 1.62	0.568
OLT area (mm^2^)			55.27 ± 21.31	
OLT volume (mm^3^)			285.56 ± 144.95	
OLT position (medial/lateral)			20/9	

Abbreviations: AOFAS, American Orthopedic Foot and Ankle Society; ATFL, anterior talofibular ligament; BMI, body mass index; CLAI, chronic lateral ankle instability; CLAI + OLT, patient with chronic lateral ankle instability and osteochondral lesion of the talus; OLT, osteochondral lesion of the talus.

### 3.2 Comparison of the Plantar Pressure Characteristic Among the Three Groups

**FIGURE 4:**
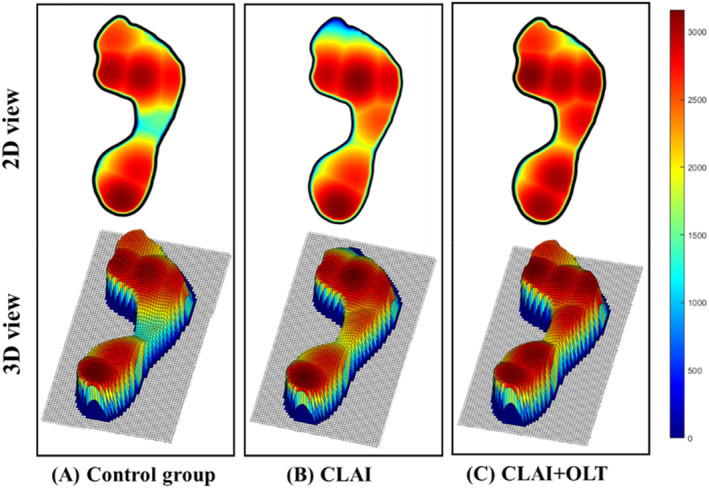
Plantar pressure distribution during single‐leg stance in the three groups: (A) the control group; (B) the CLAI group, with the affected side on the right; and (C) the CLAI + OLT group, with the affected side on the right. CLAI, chronic lateral ankle instability; CLAI + OLT, patient with chronic lateral ankle instability and osteochondral lesion of the talus.

A typical example image of the normalized plantar pressure distribution during SLS was depicted in Figure 4, and the right side was the affected side. Plantar pressure distribution during SLS were demonstrated in Table 2. Post hoc pairwise comparisons with Bonferroni correction showed that PF% in the MM region was significantly higher in the CLAI group compared to the control group (*p* = 0.028) and in the CLAI + OLT group compared to the control group (*p* = 0.001). Moreover, the patients with the CLAI group also showed increased PF% in the M3 region compared to the control group (*p* = 0.015).

**TABLE 2:** Comparison of normalized peak force across the three groups during single‐leg stance.

Parameters	Control group (*n* = 29)	CLAI (*n* = 30)	CLAI + OLT (*n* = 29)	*p* value	Bonferroni post hoc
HP (%)	17.87 ± 4.20	17.24 ± 3.67	18.10 ± 3.75	0.710	
HA (%)	9.25 ± 2.51	8.94 ± 2.74	8.92 ± 2.50	0.872	
ML (%)	10.94 ± 2.64	9.31 ± 2.94	10.51 ± 3.03	0.093	
MM (%)	0.40 ± 0.62	1.06 ± 0.82	1.39 ± 0.99	0.001*	Con < CLAI (*p* = 0.028)^a^
					Con < CLAI + OLT (*p* = 0.001)^b^
M1 (%)	12.34 ± 6.17	14.14 ± 4.05	13.33 ± 3.24	0.474	
M3 (%)	11.77 ± 5.52	15.49 ± 3.56	12.42 ± 3.83	0.012*	Con < CLAI (*p* = 0.015)^a^
M5 (%)	12.29 ± 4.53	13.81 ± 2.99	12.73 ± 4.05	0.361	
T1 (%)	7.17 ± 3.84	6.34 ± 3.30	6.97 ± 3.05	0.678	

Abbreviations: CLAI, chronic lateral ankle instability; CLAI + OLT, patient with chronic lateral ankle instability and osteochondral lesion of the talus; HA region, anterior heel; HP region, posterior heel; M1 region, first metatarsal; M3 region, third metatarsal; M5 region, fifth metatarsal; ML region, lateral midfoot; MM region, medial midfoot; T1 region, the first phalanx.

^a^ Bonferroni post hoc test was significant between the control group and the CLAI group.

^b^ Bonferroni post hoc test was significant between the control group and the CLAI + OLT group.

^*^ *p* < 0.05, ANOVA result was significant.

During SLS, significant differences in postural stability were observed among the groups. Post hoc pairwise comparisons with Bonferroni correction showed that the TTB absolute_AP were significantly lower in the CLAI + OLT group than in the control group (*p* = 0.032), whereas regarding COP outcomes, both the CLAI group (*p* = 0.039) and the CLAI + OLT group (*p* = 0.026) showed significantly greater COP variance in the AP direction on the affected side compared to the control group. The detailed TTB and COP parameters were shown in Tables 3 and 4, respectively.

**TABLE 3:** Comparison of TTB parameters across the three groups during single‐leg stance.

Parameters	Control group (*n* = 29)	CLAI (*n* = 30)	CLAI + OLT (*n* = 29)	*p* value	Bonferroni post hoc
TTB absolute_ML (s)	0.90 ± 0.50	0.80 ± 0.46	0.86 ± 0.46	0.719	
TTB absolute_AP (s)	1.38 ± 0.71	1.07 ± 0.59	0.94 ± 0.51	0.029*	Con > CLAI + OLT (*p* = 0.032)^a^
TTB mean_ML (s)	5.58 ± 3.63	6.29 ± 3.46	6.40 ± 2.63	0.609	
TTB mean_AP (s)	10.26 ± 4.37	10.14 ± 4.83	9.40 ± 3.93	0.756	
TTB SD_ML (s)	6.89 ± 4.29	9.00 ± 5.51	9.79 ± 5.00	0.092	
TTB SD_AP (s)	11.06 ± 5.26	11.86 ± 6.16	13.31 ± 7.38	0.431	

Abbreviations: AP, anterior–posterior; CLAI, chronic lateral ankle instability; CLAI + OLT, patient with chronic lateral ankle instability and osteochondral lesion of the talus; ML, medial‐lateral; SD, standard deviation; TTB, time‐to‐boundary.

^a^ Bonferroni post hoc test was significant between the control group and the CLAI + OLT group.

^*^ *p* < 0.05, ANOVA result was significant.

**TABLE 4:** Comparison of COP parameters across the three groups during single‐leg stance.

Parameters	Control group (*n* = 29)	CLAI (*n* = 30)	CLAI + OLT (*n* = 29)	*p* value	Bonferroni post hoc
Mean COP_ML (cm)	−0.29 ± 0.25	−0.13 ± 0.39	−0.12 ± 0.45	0.183	
Mean COP_AP (cm)	−1.46 ± 2.69	−0.63 ± 1.97	−1.73 ± 3.68	0.351	
COP variance_ML (cm)	0.27 ± 0.12	0.27 ± 0.11	0.30 ± 0.13	0.594	
COP variance_AP (cm)	0.43 ± 0.16	0.57 ± 0.16	0.58 ± 0.33	0.047*	Con < CLAI (*p* = 0.039)^a^
					Con < CLAI + OLT (*p* = 0.026)^b^

Abbreviations: AP, anterior–posterior; CLAI, chronic lateral ankle instability; CLAI + OLT, patient with chronic lateral ankle instability and osteochondral lesion of the talus; COP, center of pressure; ML, medial‐lateral.

^a^ Bonferroni post hoc test was significant between the control group and the CLAI group.

^b^ Bonferroni post hoc test was significant between the control group and the CLAI + OLT group.

^*^ *p* < 0.05, ANOVA result was significant.

### 3.3 Comparison of the Plantar Pressure Characteristics of the Affected and Unaffected Side

**FIGURE 5:**
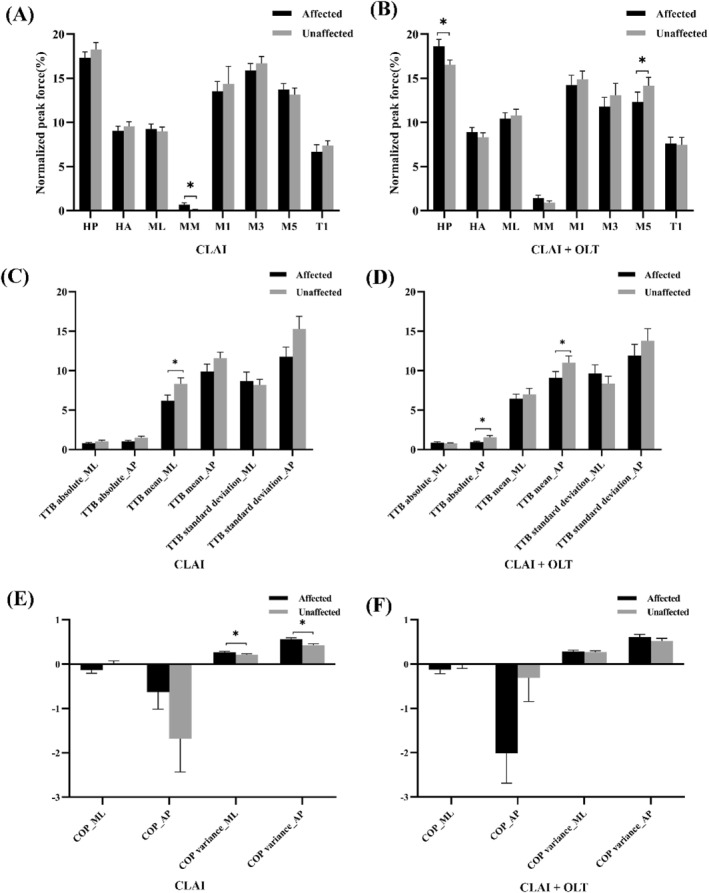
Comparison of parameters between the affected and unaffected sides in the CLAI group and the CLAI + OLT group during single‐leg stance: (A) plantar pressure parameters in CAI; (B) plantar pressure parameters in CLAI + OLT; (C) TTB parameters in CLAI; (D) TTB parameters in CLAI + OLT; (E) COP parameters in CLAI; and (F) COP parameters in CLAI + OLT. AP, anterior–posterior; CLAI, chronic lateral ankle instability; CLAI + OLT, patient with chronic lateral ankle instability and osteochondral lesion of the talus; COP, center of pressure; ML, medial‐lateral; TTB, time‐to‐boundary. *p < 0.05: significant differences between the affected sides and unaffected sides.

When the two sides were compared, in the CLAI group, normalized peak force of medial midfoot of the affected side was significantly higher than that of the unaffected side (*p* = 0.020) (Figure 5A). In the CLAI + OLT group, the normalized peak force in the HP region of the unaffected side was significantly lower than that of the affected side (*p* = 0.012), whereas the normalized peak force in the fifth metatarsal region of the unaffected side was significantly higher than that of the affected side (*p* = 0.030) (Figure 5B).

For patients with CLAI, the mean TTB in the ML direction was significantly lower on the affected side compared to the unaffected side (*p* = 0.040) (Figure 5C). For patients with CLAI + OLT, both the absolute and mean TTB in the AP direction were significantly lower on the affected side compared to the unaffected side (*p* = 0.003, 0.039) (Figure 5D).

For patients with CLAI, the variance of COP in both ML and AP directions was significantly greater on the affected side (*p* = 0.039, 0.011), indicating increased postural instability (Figure 5E). No significant differences were observed in COP parameters between the affected and unaffected sides of patients with CLAI + OLT, it might due to the stability of medial‐lateral and anterior–posterior direction of the unaffected sides were also influenced (Figure 5F).

### 3.4 Receiver Operating Characteristic (ROC) Curve Analysis

**FIGURE 6:**
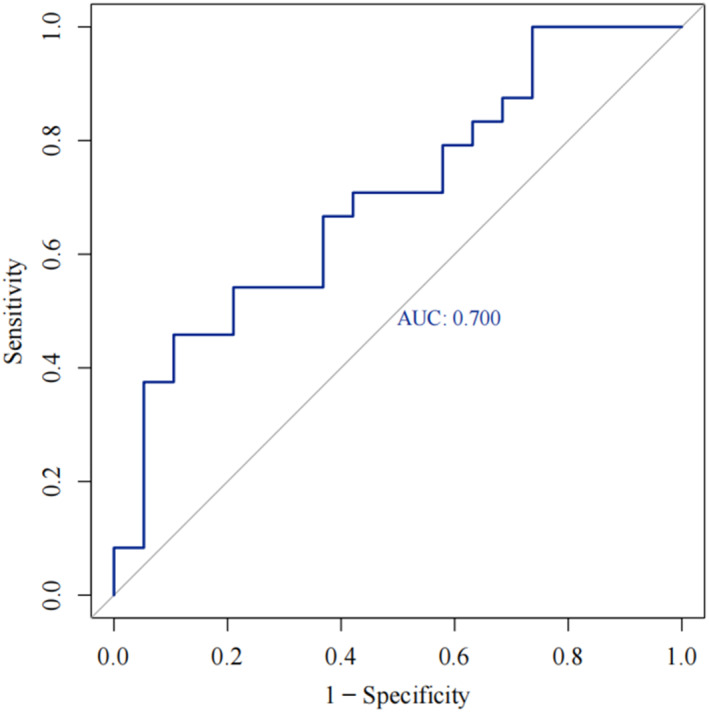
ROC curve analysis for the M3 region based on normalized peak force in the CLAI group and the CLAI + OLT group.

A ROC curve analysis was performed to delineate the clinical significance of plantar pressure features in patients (Figure 6). An area under curve (AUC) > 0.65 indicated a potential diagnostic value of a certain indicator for the disease. Normalized peak force of M3 region represented an ability to discriminate patients with CLAI and CLAI + OLT. The AUC for the M3 region was 0.700 ± 0.082, with a 95% confidence interval from 0.539 to 0.860 (*p* = 0.026), and the cut‐off point was 15.80.

### 3.5 Subgroup Analysis of Plantar Pressure Parameters

Previous studies indicated that gender, BMI, and generalized joint hypermobility have a great influence on the incidence of CLAI [41, 42]. Therefore, we carried out subgroup analysis of gender, BMI, and Beighton score.

In the male subgroup, the normalized peak force in the M3 region of the affected side was significantly higher in the CLAI group than in the CLAI + OLT group (*p* = 0.047). In the female subgroup, the normalized peak force in the T1 region was significantly lower in the CLAI group compared to the CLAI + OLT group (*p* = 0.047) (Table 5).

**TABLE 5:** Subgroup analysis of normalized peak force by gender.

Subgroups	Male			Female		
	CLAI (*n* = 18)	CLAI + OLT (*n* = 24)	*p* value	CLAI (*n* = 12)	CLAI + OLT (*n* = 5)	*p* value
HP (%)	17.50 ± 3.59	18.13 ± 3.63	0.598	16.82 ± 3.95	17.97 ± 4.91	0.659
HA (%)	7.73 ± 2.26	8.63 ± 2.42	0.248	10.76 ± 2.47	10.02 ± 2.80	0.589
ML (%)	9.04 ± 2.44	10.39 ± 3.18	0.157	9.76 ± 3.70	11.07 ± 2.48	0.528
MM (%)	0.92 ± 0.70	1.49 ± 1.04	0.137	1.49 ± 1.12	1.03 ± 0.84	0.576
M1 (%)	13.49 ± 3.07	13.46 ± 3.03	0.982	14.74 ± 4.85	12.95 ± 4.34	0.535
M3 (%)	14.79 ± 3.88	11.78 ± 3.59	0.047*	16.18 ± 3.22	14.83 ± 4.23	0.510
M5 (%)	14.40 ± 3.01	12.14 ± 3.03	0.050	13.12 ± 2.97	15.11 ± 6.99	0.423
T1 (%)	7.14 ± 3.35	6.37 ± 3.00	0.526	5.48 ± 3.17	9.25 ± 2.29	0.047*

Abbreviations: CLAI, chronic lateral ankle instability; CLAI + OLT, patient with chronic lateral ankle instability and osteochondral lesion of the talus; HA region, anterior heel; HP region, posterior heel; M1 region, first metatarsal; M3 region, third metatarsal; M5 region, fifth metatarsal; ML region, lateral midfoot; MM region, medial midfoot; T1 region, the first phalanx.

^*^ *p* < 0.05, significant differences between the CLAI group and the CLAI + OLT group.

In the BMI < 25 subgroup, the normalized peak force in the M3 region was higher in the CAI group than in the CAI + OLT group (*p* = 0.010). For the BMI > 25 subgroup, the normalized peak force in the HP region was lower in the CAI group than in the CAI + OLT group (*p* = 0.043) (Table 6).

**TABLE 6:** Subgroup analysis of normalized peak force by BMI.

Subgroups	BMI < 25			BMI ≥ 25		
	CLAI (*n* = 19)	CLAI + OLT (*n* = 14)	*p* value	CLAI (*n* = 11)	CLAI + OLT (*n* = 15)	*p* value
HP (%)	19.16 ± 2.60	19.21 ± 4.13	0.966	14.11 ± 2.99	16.88 ± 3.01	0.043*
HA (%)	9.42 ± 3.16	8.45 ± 2.67	0.373	8.12 ± 1.63	9.48 ± 2.29	0.124
ML (%)	9.58 ± 2.67	10.14 ± 2.35	0.556	8.88 ± 3.42	10.91 ± 3.72	0.197
MM (%)	0.98 ± 0.83	1.21 ± 0.86	0.613	1.14 ± 0.86	1.57 ± 1.14	0.428
M1 (%)	16.23 ± 3.61	13.90 ± 3.83	0.183	10.57 ± 1.26	12.84 ± 2.74	0.063
M3 (%)	16.95 ± 2.83	12.91 ± 4.23	0.010*	13.44 ± 3.58	11.88 ± 3.49	0.351
M5 (%)	14.68 ± 3.38	12.80 ± 5.11	0.277	12.61 ± 1.94	12.67 ± 2.92	0.961
T1 (%)	6.75 ± 3.44	6.08 ± 3.54	0.642	5.74 ± 3.17	7.97 ± 2.18	0.095

Abbreviations: CLAI, chronic lateral ankle instability; CLAI + OLT, patient with chronic lateral ankle instability and osteochondral lesion of the talus; HA region, anterior heel; HP region, posterior heel; M1 region, first metatarsal; M3 region, third metatarsal; M5 region, fifth metatarsal; ML region, lateral midfoot; MM region, medial midfoot; T1 region, the first phalanx.

^*^ *p* < 0.05, significant differences between the CLAI group and the CLAI + OLT group.

In the Beighton score < 5 subgroup, the normalized peak force in the M3 region was significantly higher in the CAI group than in the CAI + OLT group (*p* = 0.004). No significant differences were found between the two group when the Beighton score > 5 (Table 7).

**TABLE 7:** Subgroup analysis of normalized peak force by Beighton score.

Subgroups	Beighton < 5			Beighton ≥ 5		
	CLAI (*n* = 19)	CLAI + OLT (*n* = 21)	*p* value	CLAI (*n* = 11)	CLAI + OLT (*n* = 8)	*p* value
HP (%)	17.14 ± 3.83	18.01 ± 3.86	0.511	17.44 ± 3.55	18.30 ± 3.79	0.640
HA (%)	8.33 ± 2.69	9.32 ± 2.66	0.277	9.99 ± 2.62	7.96 ± 1.92	0.097
ML (%)	9.15 ± 2.92	10.56 ± 3.47	0.201	9.62 ± 3.10	10.40 ± 1.88	0.565
MM (%)	0.88 ± 0.70	1.42 ± 1.03	0.176	1.47 ± 0.99	1.33 ± 1.03	0.823
M1 (%)	14.68 ± 3.98	13.49 ± 3.75	0.479	13.55 ± 4.27	12.97 ± 1.72	0.781
M3 (%)	15.97 ± 3.63	11.75 ± 3.65	0.004*	14.81 ± 3.53	14.92 ± 3.91	0.960
M5 (%)	13.85 ± 2.76	12.27 ± 4.27	0.240	13.75 ± 3.44	14.12 ± 3.33	0.844
T1 (%)	6.20 ± 3.59	7.12 ± 3.02	0.460	6.56 ± 3.00	6.55 ± 3.46	0.996

Abbreviations: CLAI, chronic lateral ankle instability; CLAI + OLT, patient with chronic lateral ankle instability and osteochondral lesion of the talus; HA region, anterior heel; HP region, posterior heel; M1 region, first metatarsal; M3 region, third metatarsal; M5 region, fifth metatarsal; ML region, lateral midfoot; MM region, medial midfoot; T1 region, the first phalanx.

^*^ *p* < 0.05, significant differences between the CLAI group and the CLAI + OLT group.

For the CLAI + OLT group, the subgroup analysis was also carried out for location of the OLT. As shown in Table 8, there were no significant differences in the plantar pressure characteristics of OLT at different localizations (medial talus and lateral talus).

**TABLE 8:** Subgroup analysis of normalized peak force by OLT localizations.

Parameters	Medial talus (*n* = 20)	Lateral talus (*n* = 9)	*p* value
HP (%)	17.80 ± 3.61	18.66 ± 4.19	0.611
HA (%)	8.87 ± 2.49	9.02 ± 2.70	0.892
ML (%)	11.04 ± 3.39	9.51 ± 2.05	0.259
MM (%)	1.61 ± 1.06	0.98 ± 0.77	0.267
M1 (%)	12.86 ± 2.94	14.50 ± 3.98	0.357
M3 (%)	13.27 ± 3.74	10.58 ± 3.63	0.160
M5 (%)	13.13 ± 4.29	12.00 ± 3.78	0.567
T1 (%)	6.95 ± 3.28	7.01 ± 2.87	0.971

Abbreviations: HA region, anterior heel; HP region, posterior heel; M1 region, first metatarsal; M3 region, third metatarsal; M5 region, fifth metatarsal; ML region, lateral midfoot; MM region, medial midfoot; T1 region, the first phalanx.

## 4 Discussion

The application of plantar pressure distribution measurements in the diagnosis and evaluation of musculoskeletal diseases is increasing, which could improve diagnostic accuracy and informing more tailored treatment plans [43]. This study investigated the plantar pressure characteristics during SLS in patients with CLAI and those with OLT using a novel portable plantar pressure measurement system. Both patient groups showed increased MM pressure, indicating abnormal loading due to ligamentous insufficiency. Isolated patients with CLAI also displayed greater M3 pressure, a change less evident with concomitant OLT, suggesting biomechanical differences between groups. In the CLAI + OLT group, altered loading was accompanied by reduced anteroposterior stability and contralateral pressure redistribution, reflecting bilateral compensatory adaptations to unilateral dysfunction. These changes in plantar loading highlighted the value of pressure analysis as an early assessment of CLAI with OCLT. By elucidating how concomitant osteochondral lesions exacerbate plantar pressure deviations and postural instability in patients with chronic lateral ankle instability, this study deepened our understanding of the interplay between mechanical instability and cartilage pathology, while providing actionable insights for early detection strategies and rehabilitation approaches aimed at mitigating aberrant joint loading and neuromuscular control.

Compared with healthy subjects, all the patients with isolated CLAI and those with OLT showed increased plantar pressure in the MM and the patients with CLAI also showed increased plantar pressure in the M3 region. These adaptations may reflect compensatory strategies to stabilize the ankle due to impaired sensorimotor control, with patients with ankle instability shifting weight medially to compensate for reduced proprioception and weakened peroneal muscle activation [44, 45]. The increased MM loading observed in both groups aligned with recent findings by Cao et al. They reported that patients with concomitant CLAI and OLT showed increased medial shear force compared with healthy subjects during 11.3%–19.1% of the gait cycle [46]. The medial midfoot overload might increase strain on midfoot ligaments and contribute to degenerative changes in the talocrural joint. The elevated pressure at the M3 region of patients with CLAI suggested an adaptive response to prevent excessive inversion, driven by lateral ligament insufficiency and requiring increased activation of the tibialis anterior and flexor digitorum longus to stabilize the ankle. This aligned with studies, such as Huang et al., which demonstrated significantly higher peak pressures in the M3 region during running and lateral shuffling in athletes with ankle instability [47], and Zhang et al., who observed altered plantar pressure with increased lateral forefoot loading and decreased medial foot pressure during backward walking in patients with CLAI [22]. Neuromuscular adaptations likely contributed to these pressure changes. Previous studies reported that the patients with CLAI showed reduced tibialis anterior activation accompanied by compensatory increases in peroneus longus and gluteus medius activity in patients with CLAI [44, 48]. Hou ZC also reported that the initial inversion muscle strength weakness of patients with CLAI might be the potential risk factors of sprain recurrence after balance training [49]. These findings not only reflected maladaptive neuromuscular strategies but also served as precursors to progressive joint degeneration.

The observed reductions in TTB and increased COP trajectory in patients with concomitant CLAI and OLT during SLS suggested a significant impairment in their ability to maintain stability in the anterior–posterior direction. TTB and COP are well‐established indicators of postural stability, with the former reflecting the time available to correct a destabilizing perturbation and the latter providing insight into the shifting of weight during stance. This study was consistent with previous studies. Meredith Pope et al. found that individuals with CAI exhibited significant impairments in spatial postural control during SLS, characterized by increased COP sway and decreased stability compared to healthy controls [50]. Masafumi Terada et al. found that the TTB in the ML and AP direction of the patients with CLAI were smaller than those of the normal controls during a single‐leg balance task with eyes‐closed [51]. Abe et al. also demonstrated altered COP distribution biased toward the anterolateral foot and increased instability during SLS in individuals with ankle sprain history using force plates combined with head and foot accelerometry [21]. Further corroborating these results, other studies revealed that patients with CLAI display decreased medial‐lateral TTB and a more lateral COP during dynamic tasks such as walking and running stance phase [19, 20, 22]. However, up to now, there were no studies on the stability of OLT patients during SLS. Recent biomechanical investigations provided mechanistic insight into the stability deficits observed in patients with concomitant CLAI and OLT. Torp et al. reported significant associations between walking plantar pressure patterns and regional cartilage variations, whereas Yu et al. demonstrated that regional plantar force characteristics in patients with CLAI correlate with subtle but functionally relevant changes in foot and talar geometry [14, 40]. OLT often causes irregularities at the talar articular surface and can significantly disrupt the proprioceptive feedback mechanisms essential for effective ankle joint control, thus hindering the motor control required for stable postural control [12, 52]. Moreover, OLT‐induced changes to the talus can exacerbate pain and functional limitations, further diminishing the patient's capacity to generate effective neuromuscular responses for postural adjustments.

In patients with unilateral concomitant CLAI and OLT, abnormal plantar pressure distribution extended beyond the affected limb to involve the contralateral side. Specifically, the unaffected limb showed decreased rearfoot loading, increased pressure in the M5 region, and an anterior shift of the center of pressure. This bilateral plantar pressure alteration was similar to the findings of recent findings. A study demonstrated that unilateral patients with CAI exhibited increased lateral plantar pressure and elevated COP velocity on both affected and unaffected sides [19]. Lee et al. reported that the unaffected side in recurrent ankle sprain patients showed significantly worse static and dynamic postural stability, along with deficits in neuromuscular control across inversion, eversion, dorsiflexion, and plantarflexion [45]. These phenomenons may be attributed to altered central control during activities, reflecting a broader impairment in neuromuscular control and postural stability. The healthy limb underwent functional compensation, with the body employing compensatory postural control strategies to maintain overall stability [19, 45]. Therefore, rehabilitation for patients with concomitant CLAI and OLT should not only focus on the affected side but also incorporate functional training for both lower limbs to restore balance and reduce compensatory patterns [53].

This research disclosed that the shoe‐integrated sensor system had a moderate rate of accuracy in differentiating patients with CLAI with and without OLT injuries. With ROC analysis showing the M3 region peak pressure yielding an AUC of 0.700 (*p* = 0.026), sensitivity of 45.8%, and specificity of 89.5%. These findings aligned with previous research supporting plantar pressure assessment as a valuable tool for disease diagnosis and prognostic evaluation. Our previous study found that the shoe‐integrated sensor system demonstrated remarkable precision in identifying concomitant syndesmotic injuries in patients with chronic lateral ankle instability, achieving 80% sensitivity and 75% specificity through evaluation of plantar pressure ratios during walking [25]. Wilzman et al. found that higher plantar loading during running, walking, and athletic movements could predict the risk of future bone stress injury in elite collegiate runners [54]. It was known that there was a significant correlation between sex, weight, joint relaxation, and plantar pressure distribution for patients with CLAI, so further subgroup analysis was conducted [49]. In this study, the subgroup analysis further highlighted that patients with CLAI with male sex, BMI < 25, and Beighton < 5 showed significantly increased pressure in the M3 region. This suggested that individuals with these characteristics may rely more on the lateral forefoot to maintain balance, which was critical for developing targeted interventions for CLAI. Additionally, when BMI ≥ 25, patients with concomitant CLAI and OLT exhibited a significant increase in pressure in the HP region. This demonstrated that patients with high‐BMI may rely more on the rearfoot due to increased weight‐bearing demands, reflecting enhanced dependence on the posterior foot for postural control [20]. Understanding these subgroup‐specific plantar pressure characteristics can help to adopt more precise and individualized treatment strategies for patients with CLAI with or without OLT.

This study provided critical insights into the functional assessment and rehabilitation of patients with CLAI with or without OLT. By analyzing plantar pressure characteristics during SLS, we could more accurately assess postural control capabilities and gain insights into the progression of cartilage damage. This information was crucial for developing tailored rehabilitation strategies. It is necessary to take targeted treatments to elevate peroneal activation level and thus decrease medial loading of the talus for patients with isolated CLAI and those with OLT. Interventions, such as ankle joint mobilizations, plantar fascia massage, and gastrocnemius stretching, have been shown to improve medial and lateral foot pressure distribution [55]. Strengthening exercises targeting both medial and lateral muscles, along with balance training, could also enhance postural control [49]. In particular, patients with CLAI might benefit from focused training to improve the stability of the lateral forefoot. For patients with concomitant CLAI and OLT, rehabilitation should target both affected and unaffected sides to prevent secondary injuries, especially for those with BMI ≥ 25, with an emphasis on rearfoot load control. If necessary, a Broström‐Gould surgery can be performed to provide more persistent recovery of stability for patients with CLAI [56].

To our knowledge, this study was the first to compare the plantar pressure characteristics during SLS between patients with CLAI and those with concomitant CLAI and OLT, which could provide a deeper understanding of the biomechanical alterations in patients with CLAI, contributing valuable insights into the progression of OLT. Moreover, by utilizing portable pressure insoles for data collection, we significantly enhanced the real‐time accuracy and precision of plantar pressure measurements, allowing for more practical and natural assessments in clinical settings [57]. This study also has certain limitations. First, although the total sample size exceeded the calculated minimum, subgroups still had limited participants. These results should be considered exploratory, and future investigation with a larger sample size is needed to obtain more definitive conclusions. Second, the inclusion of a broad age range may have introduced substantial variability in musculoskeletal biomechanics. This age range was selected to maintain the study's exploratory nature and to offer preliminary insights across diverse demographics. Future research should consider age stratification to achieve more precise and targeted conclusions.

## 5 Conclusions

Increased medial midfoot plantar pressure was found in patients with CLAI, with further pronounced anteroposterior instability and contralateral pressure abnormalities noted in patients with CLAI with OLT. Monitoring plantar pressure in the third metatarsal region could provide valuable insights for the early warning and management of CLAI and OLT. Designing specific rehabilitation strategies aimed at improving pathological loading patterns and stability might be an effective strategy to prevent long‐term joint degeneration of these patients.

## Author Contributions

**Ting Zhu:** conceptualization, formal analysis, writing – original draft. **Rui Chen:** investigation, writing – original draft. **Haoyang Kang:** investigation. **Fangyuan Ding:** formal analysis. **Rui Guo:** methodology, resources. **Xiaoming Wu:** methodology, resources. **Dong Jiang:** conceptualization, supervision, writing – review and editing.

## Funding

This study was supported by Capital's Funds for Health Improvement and Research (Grant 2022‐2Z‐40913).

## Ethics Statement

This work was approved by the Medical Ethics Committee of Peking University Third Hospital (M2024712) and all participants agreed and signed the informed consent.

## Consent

Informed consent was obtained from all individual participants included in the study.

## Conflicts of Interest

The authors declare no conflicts of interest.

## Data Availability

The dataset supporting the conclusions of this article is available from the corresponding author upon request.

## References

[1] N. A. Ferran and N. Maffulli , “Epidemiology of Sprains of the Lateral Ankle Ligament Complex,” Foot and Ankle Clinics 11, no. 3 (2006): 659–662, 10.1016/j.fcl.2006.07.002. 16971255

[2] C. Doherty , E. Delahunt , B. Caulfield , J. Hertel , J. Ryan , and C. Bleakley , “The Incidence and Prevalence of Ankle Sprain Injury: A Systematic Review and Meta‐Analysis of Prospective Epidemiological Studies,” Sports Medicine 44, no. 1 (2014): 123–140, 10.1007/s40279-013-0102-5. 24105612

[3] M. M. Herzog , Z. Y. Kerr , S. W. Marshall , and E. A. Wikstrom , “Epidemiology of Ankle Sprains and Chronic Ankle Instability,” Journal of Athletic Training 54, no. 6 (2019): 603–610, 10.4085/1062-6050-447-17. 31135209 PMC6602402

[4] C. Thompson , S. Schabrun , R. Romero , A. Bialocerkowski , J. van Dieen , and P. Marshall , “Factors Contributing to Chronic Ankle Instability: A Systematic Review and Meta‐Analysis of Systematic Reviews,” Sports Medicine 48, no. 1 (2018): 189–205, 10.1007/s40279-017-0781-4. 28887759

[5] D. Y. Wang , C. Jiao , Y. F. Ao , et al., “Risk Factors for Osteochondral Lesions and Osteophytes in Chronic Lateral Ankle Instability: A Case Series of 1169 Patients,” Orthopaedic Journal of Sports Medicine 8, no. 5 (2020): 2325967120922821, 10.1177/2325967120922821. 32518802 PMC7252382

[6] W. J. Choi , J. W. Lee , S. H. Han , B. S. Kim , and S. K. Lee , “Chronic Lateral Ankle Instability: The Effect of Intra‐Articular Lesions on Clinical Outcome,” American Journal of Sports Medicine 36, no. 11 (2008): 2167–2172, 10.1177/0363546508319050. 18669983

[7] E. J. Wijnhoud , Q. G. H. Rikken , J. Dahmen , I. N. Sierevelt , S. A. S. Stufkens , and G. Kerkhoffs , “One in Three Patients With Chronic Lateral Ankle Instability Has a Cartilage Lesion,” American Journal of Sports Medicine 51, no. 7 (2023): 1943–1951, 10.1177/03635465221084365. 35384745 PMC10240649

[8] E. A. Wikstrom , T. Hubbard‐Turner , and P. O. McKeon , “Understanding and Treating Lateral Ankle Sprains and Their Consequences: A Constraints‐Based Approach,” Sports Medicine 43, no. 6 (2013): 385–393, 10.1007/s40279-013-0043-z. 23580392

[9] K. J. Hunt , A. T. Lee , D. P. Lindsey , W. Slikker , and L. B. Chou , “Osteochondral Lesions of the Talus: Effect of Defect Size and Plantarflexion Angle on Ankle Joint Stresses,” American Journal of Sports Medicine 40, no. 4 (2012): 895–901, 10.1177/0363546511434404. 22366518

[10] O. E. Yausep , I. Madhi , and D. Trigkilidas , “Platelet Rich Plasma for Treatment of Osteochondral Lesions of the Talus: A Systematic Review of Clinical Trials,” Journal of Orthopaedics 18 (2020): 218–225, 10.1016/j.jor.2020.01.046. 32071508 PMC7013135

[11] M. Mortezanejad , Z. Ebrahimabadi , A. Rahimi , A. Maleki , A. A. Baghban , and F. Ehsani , “Postural Adjustment and Muscle Activity During Each Phase of Gait Initiation in Chronic Ankle Instability: An Observational Study,” BMC Sports Science, Medicine & Rehabilitation 16, no. 1 (2024): 248, 10.1186/s13102-024-01033-x. PMC11657741 39695841

[12] Z. C. Hou , X. Miao , Y. F. Ao , et al., “Characteristics and Predictors of Muscle Strength Deficit in Mechanical Ankle Instability,” BMC Musculoskeletal Disorders 21, no. 1 (2020): 730, 10.1186/s12891-020-03754-9. 33172443 PMC7654059

[13] S. Cao , C. Wang , S. Jiang , et al., “Concomitant Osteochondral Lesions of the Talus Affect the Stair Descent Biomechanics of Patients With Chronic Ankle Instability: A Pilot Study,” Gait & Posture 96 (2022): 306–313, 10.1016/j.gaitpost.2022.06.009. 35772346

[14] D. M. Torp , A. C. Thomas , T. Hubbard‐Turner , and L. Donovan , “Plantar Pressure Profile During Walking Is Associated With Talar Cartilage Characteristics in Individuals With Chronic Ankle Instability,” Clinical Biomechanics 95 (2022): 105656, 10.1016/j.clinbiomech.2022.105656. 35504121

[15] Z. Chen , X. Xue , Q. Li , et al., “Outcomes of a Novel All‐Inside Arthroscopic Anterior Talofibular Ligament Repair for Chronic Ankle Instability,” International Orthopaedics 47, no. 4 (2023): 995–1003, 10.1007/s00264-023-05721-0. 36790535

[16] T. Su , X. Cheng , Y. Zhu , et al., “Patients With Chronic Lateral Ankle Instability and Small Osteochondral Lesions of the Talus Obtain Good Postoperative Results: A Minimum 10‐Year Follow‐Up With Radiographic Evidence,” Foot & Ankle International 46, no. 3 (2025): 277–286, 10.1177/10711007241311858. 39868597

[17] R. Guo , X. Cheng , Z. C. Hou , et al., “A Shoe‐Integrated Sensor System for Long‐Term Center of Pressure Evaluation,” IEEE Sensors Journal 21, no. 23 (2021): 27037–27044, 10.1109/jsen.2021.3116249.

[18] S. Speight , S. Reel , and J. Stephenson , “Can the F‐Scan in‐Shoe Pressure System Be Combined With the GAITRite® Temporal and Spatial Parameter‐Recording Walkway as a Cost‐Effective Alternative in Clinical Gait Analysis? A Validation Study,” Journal of Foot and Ankle Research 16, no. 1 (2023): 30, 10.1186/s13047-023-00627-x. 37194058 PMC10186786

[19] D. P. Wan , H. L. Bao , J. P. Wang , et al., “Plantar Pressure Distribution and Posture Balance During Walking in Individuals With Unilateral Chronic Ankle Instability: An Observational Study,” Medical Science Monitor: International Medical Journal of Experimental and Clinical Research 29 (2023): e940252, 10.12659/msm.940252. 37340627 PMC10291896

[20] K. E. Morrison , D. J. Hudson , I. S. Davis , et al., “Plantar Pressure During Running in Subjects With Chronic Ankle Instability,” Foot & Ankle International 31, no. 11 (2010): 994–1000, 10.3113/fai.2010.0994. 21189193

[21] Y. Abe , T. Sugaya , and M. Sakamoto , “Postural Control Characteristics During Single Leg Standing of Individuals With a History of Ankle Sprain: Measurements Obtained Using a Gravicorder and Head and Foot Accelerometry,” Journal of Physical Therapy Science 26, no. 3 (2014): 447–450, 10.1589/jpts.26.447. 24707105 PMC3976024

[22] L. Zhang , T. Liu , X. Zhou , et al., “Gait Characteristics and Deviation Factors of Backward Walking in Patients With Chronic Ankle Instability,” Sport Health 17, no. 4 (2024): 815–823, 10.1177/19417381241277804. PMC11556632 39279244

[23] M. R. Powell , C. J. Powden , M. N. Houston , and M. C. Hoch , “Plantar Cutaneous Sensitivity and Balance in Individuals With and Without Chronic Ankle Instability,” Clinical Journal of Sport Medicine 24, no. 6 (2014): 490–496, 10.1097/jsm.0000000000000074. 24451692

[24] P. A. Gribble , E. Delahunt , C. M. Bleakley , et al., “Selection Criteria for Patients With Chronic Ankle Instability in Controlled Research: A Position Statement of the International Ankle Consortium,” Journal of Athletic Training 49, no. 1 (2014): 121–127, 10.4085/1062-6050-49.1.14. 24377963 PMC3917288

[25] Y. Li , R. Guo , Y. Wang , et al., “Shoe‐Integrated Sensor System for Diagnosis of the Concomitant Syndesmotic Injury in Chronic Lateral Ankle Instability: A Prospective Double‐Blind Diagnostic Test,” Nanomaterials (Basel) 13, no. 9 (2023): 1539, 10.3390/nano13091539. 37177084 PMC10180214

[26] W. Liu , H. Li , and Y. Hua , “Quantitative Magnetic Resonance Imaging (MRI) Analysis of Anterior Talofibular Ligament in Lateral Chronic Ankle Instability Ankles Pre‐ and Postoperatively,” BMC Musculoskeletal Disorders 18, no. 1 (2017): 397, 10.1186/s12891-017-1758-z. 28899377 PMC5596477

[27] S. M. Choi , B. K. Cho , and S. H. Kim , “The Influence of Suture‐Tape Augmentation on Biological Healing of the Anterior Talofibular Ligament in Chronic Ankle Instability: A Quantitative Analysis Using MRI,” Journal of Foot and Ankle Surgery 61, no. 5 (2022): 957–963, 10.1053/j.jfas.2021.12.020. 35016831

[28] X. Cheng , J. Li , M. Pei , et al., “Medial Cystic Osteochondral Lesions of the Talus Exhibited Lower Sports Levels, Higher Cyst Presence Rate, and Inferior Radiological Outcomes Compared With Lateral Lesions Following Arthroscopic Bone Marrow Stimulation,” Arthroscopy: The Journal of Arthroscopic & Related Surgery 41, no. 1 (2025): 110–118, 10.1016/j.arthro.2024.05.011. 38797503

[29] Y. B. Li , X. Z. Fan , G. J. Zhou , et al., “The Severity of Preoperative Bone Marrow Oedema Negatively Influences Short‐Term Clinical Outcomes Following Arthroscopic Bone Marrow Stimulation for Osteochondral Lesions of the Talus,” Knee Surgery, Sports Traumatology, Arthroscopy 32, no. 9 (2024): 2440–2451, 10.1002/ksa.12355. 39010713

[30] P. Beighton , L. Solomon , and C. L. Soskolne , “Articular Mobility in an African Population,” Annals of the Rheumatic Diseases 32, no. 5 (1973): 413–418, 10.1136/ard.32.5.413. 4751776 PMC1006136

[31] H. B. Kitaoka , I. J. Alexander , R. S. Adelaar , J. A. Nunley , M. S. Myerson , and M. Sanders , “Clinical Rating Systems for the Ankle‐Hindfoot, Midfoot, Hallux, and Lesser Toes,” Foot & Ankle International 15, no. 7 (1994): 349–353, 10.1177/107110079401500701. 7951968

[32] M. Karahan , M. Özcan , and B. S. Cığalı , “Balance Evaluation and Gait Analysis After Arthroscopic Partial Meniscectomy,” Indian Journal of Orthopaedics 56, no. 7 (2022): 1199–1205, 10.1007/s43465-022-00621-8. 35813534 PMC9232682

[33] A. Arceri , A. Mazzotti , F. Sgubbi , et al., “Plantar Pressure Distribution in Charcot–Marie–Tooth Disease: A Systematic Review,” Sensors 25, no. 14 (2025): 4312, 10.3390/s25144312. 40732443 PMC12299274

[34] M. Nyska , S. Shabat , A. Simkin , M. Neeb , Y. Matan , and G. Mann , “Dynamic Force Distribution During Level Walking Under the Feet of Patients With Chronic Ankle Instability,” British Journal of Sports Medicine 37, no. 6 (2003): 495–497, 10.1136/bjsm.37.6.495. 14665586 PMC1724712

[35] R. Li , X. Sun , S. Yan , et al., “Recovery of the Foot Loading Patterns of Children With Excess Weight After Losing Weight: A 3‐Year Longitudinal Study,” Children 9, no. 5 (2022): 595, 10.3390/children9050595. 35626770 PMC9139758

[36] B. Chuckpaiwong , J. A. Nunley , N. A. Mall , and R. M. Queen , “The Effect of Foot Type on In‐Shoe Plantar Pressure During Walking and Running,” Gait & Posture 28, no. 3 (2008): 405–411, 10.1016/j.gaitpost.2008.01.012. 18337103

[37] M. Majlesi , “Identifying the Most Sensitive COP Variable in Static Balance: The Impact of Chronic Ankle Instability on Postural Stability,” Journal of Foot and Ankle Research 18, no. 3 (2025): e70072, 10.1002/jfa2.70072. 40750356 PMC12316512

[38] J. Hertel and L. C. Olmsted‐Kramer , “Deficits in Time‐to‐Boundary Measures of Postural Control With Chronic Ankle Instability,” Gait & Posture 25, no. 1 (2007): 33–39, 10.1016/j.gaitpost.2005.12.009. 16446093

[39] M. Oh , H. Lee , S. Han , and J. T. Hopkins , “Differences in Postural Control and Muscle Activation in Individuals With Bilateral and Unilateral Chronic Ankle Instability,” Gait & Posture 122 (2025): 255–259, 10.1016/j.gaitpost.2025.07.332. 40753867

[40] P. Yu , X. Cen , L. Xiang , et al., “Regional Plantar Forces and Surface Geometry Variations of a Chronic Ankle Instability Population Described by Statistical Shape Modelling,” Gait & Posture 106 (2023): 11–17, 10.1016/j.gaitpost.2023.08.007. 37611480

[41] Q. Liu , B. Lin , Z. Guo , Z. Ding , K. Lian , and D. Lin , “Shapes of Distal Tibiofibular Syndesmosis Are Associated With Risk of Recurrent Lateral Ankle Sprains,” Scientific Reports 7, no. 1 (2017): 6244, 10.1038/s41598-017-06602-4. 28740251 PMC5524756

[42] C. I. Lin , F. Mayer , and P. M. Wippert , “The Prevalence of Chronic Ankle Instability in Basketball Athletes: A Cross‐Sectional Study,” BMC Sports Science, Medicine and Rehabilitation 14, no. 1 (2022): 27, 10.1186/s13102-022-00418-0. PMC8857785 35180889

[43] A. Arzehgar , R. Nia , M. Hoseinkhani , F. Masoumi , S. H. Sayyed‐Hosseinian , and S. Eslami , “An Overview of Plantar Pressure Distribution Measurements and Its Applications in Health and Medicine,” Gait & Posture 117 (2025): 235–244, 10.1016/j.gaitpost.2024.12.022. 39793245

[44] R. M. Koldenhoven , M. A. Feger , J. J. Fraser , S. Saliba , and J. Hertel , “Surface Electromyography and Plantar Pressure During Walking in Young Adults With Chronic Ankle Instability,” Knee Surgery, Sports Traumatology, Arthroscopy 24, no. 4 (2016): 1060–1070, 10.1007/s00167-016-4015-3. 26856315

[45] J. H. Lee , S. H. Lee , G. W. Choi , H. W. Jung , and W. Y. Jang , “Individuals With Recurrent Ankle Sprain Demonstrate Postural Instability and Neuromuscular Control Deficits in Unaffected Side,” Knee Surgery, Sports Traumatology, Arthroscopy 28, no. 1 (2020): 184–192, 10.1007/s00167-018-5190-1. 30291398

[46] S. Cao , Y. Chen , Y. Zhu , et al., “Concomitant Osteochondral Lesion of the Talus Affects in Vivo Ankle Kinetics in Patients With Chronic Ankle Instability,” Bone & Joint Research 13, no. 12 (2024): 716–724, 10.1302/2046-3758.1312.bjr-2023-0217.r2. 39626698 PMC11614499

[47] P. Y. Huang , C. F. Lin , L. C. Kuo , and J. C. Liao , “Foot Pressure and Center of Pressure in Athletes With Ankle Instability During Lateral Shuffling and Running Gait,” Scandinavian Journal of Medicine & Science in Sports 21, no. 6 (2011): e461–e467, 10.1111/j.1600-0838.2011.01367.x. 22092510

[48] G. W. Lee , J. Lee , S. W. Shin , and J. Kim , “Quantification of Gait Characteristics and Muscle Activation in Patients With Chronic Ankle Instability During Walking on Sand: A Randomized Crossover Trial,” Medicine 103, no. 50 (2024): e40902, 10.1097/md.0000000000040902. 39686483 PMC11651517

[49] Z. C. Hou , H. S. Huang , Y. F. Ao , et al., “The Effectiveness and Sustainability of Supervised Balance Training in Chronic Ankle Instability With Grade III Ligament Injury: A One‐Year Prospective Study,” Journal of Foot and Ankle Research 15, no. 1 (2022): 9, 10.1186/s13047-022-00514-x. 35105372 PMC8805278

[50] M. Pope , L. Chinn , D. Mullineaux , P. O. McKeon , L. Drewes , and J. Hertel , “Spatial Postural Control Alterations With Chronic Ankle Instability,” Gait & Posture 34, no. 2 (2011): 154–158, 10.1016/j.gaitpost.2011.04.012. 21600773

[51] M. Terada , M. Beard , S. Carey , et al., “Nonlinear Dynamic Measures for Evaluating Postural Control in Individuals With and Without Chronic Ankle Instability,” Motor Control 23, no. 2 (2019): 243–261, 10.1123/mc.2017-0001. 30318988

[52] R. De Ridder , T. Willems , J. Vanrenterghem , and P. Roosen , “Influence of Balance Surface on Ankle Stabilizing Muscle Activity in Subjects With Chronic Ankle Instability,” Journal of Rehabilitation Medicine 47, no. 7 (2015): 632–638, 10.2340/16501977-1970. 26035531

[53] M. Bastien , H. Moffet , L. J. Bouyer , M. Perron , L. J. Hébert , and J. Leblond , “Alteration in Global Motor Strategy Following Lateral Ankle Sprain,” BMC Musculoskeletal Disorders 15, no. 1 (2014): 436, 10.1186/1471-2474-15-436. 25515309 PMC4300726

[54] A. R. Wilzman , A. S. Tenforde , K. L. Troy , et al., “Medical and Biomechanical Risk Factors for Incident Bone Stress Injury in Collegiate Runners: Can Plantar Pressure Predict Injury?,” Orthopaedic Journal of Sports Medicine 10, no. 6 (2022): 23259671221104793, 10.1177/23259671221104793. 35734769 PMC9208063

[55] P. O. McKeon and E. A. Wikstrom , “The Effect of Sensory‐Targeted Ankle Rehabilitation Strategies on Single‐Leg Center of Pressure Elements in Those With Chronic Ankle Instability: A Randomized Clinical Trial,” Journal of Science and Medicine in Sport 22, no. 3 (2019): 288–293, 10.1016/j.jsams.2018.08.017. 30244979

[56] Z. Hou , S. Ren , Y. Hu , et al., “Comparison of Subjective and Biomechanical Outcomes Between Proprioceptive Training and Modified Broström‐Gould Surgery for Chronic Ankle Instability: A Randomized Controlled Trial,” Orthopaedic Journal of Sports Medicine 12, no. 9 (2024): 23259671241274138, 10.1177/23259671241274138. 39291124 PMC11406622

[57] M. Liu , L. Guo , J. Lin , et al., “Study on the Balance and Gait Characteristics of Subjects With Generalized Joint Hypermobility Residing in High‐Altitude Using Wearable Devices: A Cross‐Sectional Study,” BMC Musculoskeletal Disorders 25, no. 1 (2024): 837, 10.1186/s12891-024-07883-3. 39438828 PMC11495022

